# Distinct Effects of Interleukin-1β Inhibition upon Cytokine Profile in Patients with Adult-Onset Still’s Disease and Active Articular Manifestation Responding to Canakinumab

**DOI:** 10.3390/jcm10194400

**Published:** 2021-09-26

**Authors:** Khetam Ghannam, Jan Zernicke, Claudia Kedor, Joachim Listing, Gerd-R. Burmester, Dirk Foell, Eugen Feist

**Affiliations:** 1 Department of Rheumatology and Clinical Immunology, Charité—Universitätsmedizin Berlin, 10117 Berlin, Germany; jan.zernicke@charite.de (J.Z.); claudia.kedor@charite.de (C.K.); gerd.burmester@charite.de (G.-R.B.); Eugen.Feist@helios-gesundheit.de (E.F.); 2 Epidemiology Unit, German Rheumatism Research Centre, 10117 Berlin, Germany; j.listing@posteo.de; 3 Pediatric Rheumatology and Immunology, University Hospital Muenster, 48149 Muenster, Germany; dfoell@uni-muenster.de; 4 Helios Department for Rheumatology Vogelsang-Gommern GmbH, 39245 Gommern, Germany

**Keywords:** adult-onset Still’s disease, canakinumab, cytokines

## Abstract

Adult-onset Still’s disease (AOSD) is a systemic auto-inflammatory disease characterized by the presence of immunologically mediated inflammation and deficient resolution of inflammation. Canakinumab is an approved IL-1β inhibitor in the treatment of AOSD with a balanced efficacy and safety profile. Since inflammatory cytokines play a major role in the pathogenesis of AOSD, we investigated the effects of canakinumab on the cytokine profile of AOSD patients from a randomized controlled trial. Multiplex analysis and ELISA were used to test the concentrations of several cytokines at three time points—week 0 (baseline), week 1 and week 4—in two patient groups—placebo and canakinumab. Two-way repeated-measures analysis of variance revealed a significant temporal effect on the concentrations of MRP 8/14, S100A12, IL-6 and IL-18 with a significant decrease at week 4 in the canakinumab group exclusively. Comparing responders with non-responders to canakinumab showed a significant decrease in MRP 8/14, IL-1RA, IL-18 and IL-6 in responders at week 4, while S100A12 levels decreased significantly in responders and non-responders. In summary, canakinumab showed a striking effect on the cytokine profile in patients with AOSD, exhibiting a clear association with clinical response.

## 1. Introduction

Adult-onset Still’s disease (AOSD) is a rare multi-systemic auto-inflammatory disease of unknown etiology, which commonly affects young adults. It is characterized by a high spiking fever, macular and salmon-colored rash, arthritis, sore throat, neutrophilic leukocytosis and hyperferritinemia [1]. AOSD was first defined by Bywaters in 1971 [2] in fourteen patients presenting with clinical manifestations very similar to childhood-onset Still’s disease, described a century ago by Sir Still, today called systemic juvenile idiopathic arthritis (sJIA) [3]. Although the etiology of AOSD remains largely elusive, there is evidence that various mechanisms contribute to the pathogenesis of AOSD; genetic susceptibility is considered as a main contributor to AOSD. Associations with distinct HLA alleles including HLA-Bw35, Cw4, DR4, DRw6, B17, B18, B35, and DR2 have been described in different ethnic groups [4,5,6]. Infections [7,8,9], as well as other immunological stimuli and a deficient resolution of inflammation, have also been implicated in the pathogenesis of AOSD [10].

Activation and amplification of inflammation are mainly driven by innate immune cells, with macrophage and neutrophil activation representing the hallmark of AOSD pathogenesis. However, adaptive immune cells including natural killer (NK) cells and T cells are also reported to be involved in the inflammatory amplification [11]. Macrophage-colony stimulating factor and interferon-γ (IFN-γ), as biomarkers reflecting macrophage activation, are both increased in patients with AOSD and correlated with disease activity [12,13]. Most circulating leukocytes expressed L-selectin, which is a cell adhesion molecule involved in their rolling on inflamed vascular endothelium prior to transmigration and is, therefore, of interest in this context [14].

The innate immune response in macrophages and neutrophils in AOSD starts with danger or pathogen signals, termed pathogen-associated molecule patterns (PAMPs) and endogenous damage-associated molecular patterns (DAMPs). Several DAMPs including high-mobility group box-1, advanced glycation end products, S100 proteins, soluble CD163, macrophage migration inhibitory factor (MIF), and neutrophil extracellular traps have been well described in the pathogenesis of AOSD [15]. These danger signals are transmitted to macrophages and neutrophils via specific Toll-like receptors and activate NACHT, LRR, and PYD domains-containing protein 3 (NLRP3) inflammasome. The NLRP3 inflammasome is a complex of proteins that activates caspase-1 activity, leading to the proteolytic cleavage of pro-interleukin (IL)-1 and IL-18 to its bioactive and mature forms [16,17,18]. IL-1β and IL-18 play a central role in AOSD pathophysiology and further promote immune cells to produce a large amount of pro-inflammatory cytokines, including IL-6, IL-8, IL-17, and tumor necrosis factor (TNF)-α, type I interferons (IFN-α and IFN-β), as well as IL-1β and IL-18 themselves, leading to an amplified inflammatory response. Moreover, macrophage activation leads to increased release of ferritin as a common marker of disease activity in Still’s disease, including AOSD [19]. The role of adaptive immune cells in the pathogenesis of AOSD was illustrated by different studies, contributing to the activation of macrophages and neutrophils and induction of IFN-γ and IL-17 [11,20]. Moreover, activation of dendritic cells through Toll-like receptor (TLR)-7 induced Th17 response and neutrophil recruitment in AOSD patients [21]. Notably, as a marker of T cell activation, soluble Interleukin-2 Receptor (sIL-2R) was also reported as a potential marker of disease activity in AOSD [22,23].

In addition to inflammatory amplification, a deficiency in the resolution of inflammation has been suggested to play a role in the pathogenesis of AOSD. Besides a deficiency of NK cells, diminished circulating regulatory T cells (Treg) were described in AOSD [24]. However, the levels of the immune-suppressive cytokine IL-10 were elevated in the serum of AOSD and correlated with disease activity [25]. Additionally, several chemokines including C-X-C motif chemokine ligands 9 (CXCL 9) MIG, (CXCL10) IP-10, and CXCL13 have been found to be potential biomarkers in AOSD [10].

Previously, IL-1, IL-6 and TNF-α antagonists have been used as biologic therapies in patients with AOSD refractory to corticosteroids or conventional disease-modifying antirheumatic drugs (c-DMARDs) in clinical practice, but results from controlled clinical trials were missing [26]. Evidence of effectiveness was available for anakinra in particular [27], as an IL-1 receptor antagonist, as well as for canakinumab, as a human antibody against IL-1β, in refractory patients with AOSD [28]. At present, both drugs are approved by the European Medicines Agency (EMA) for sJIA and AOSD with a beneficial efficacy–safety profile [29]. Canakinumab has also been approved by the Food and Drug Administration (FDA) for treatment of sJIA and AOSD. Furthermore, canakinumab is also approved for autoinflammatory periodic fever syndromes including cryopyrin-associated periodic syndrome (CAPS), tumor necrosis factor receptor-associated periodic syndrome (TRAPS), mevalonate kinase deficiency (MKD) and familial Mediterranean fever (FMF) [29]. Since no predictive or associated markers for response to IL1 inhibitors have been established in patients with AOSD so far, this study investigated the cytokine profile in AOSD patients in more detail. To correlate biomarkers to outcome, we used samples from the only randomized controlled trial (RCT) with canakinumab performed so far [30].

## 2. Patients

Patient materials were taken from the phase II Canakinumab for Treatment of Adult-Onset Still’s Disease to Achieve Reduction of Arthritic Manifestation (CONSIDER) study, which was performed as a multicenter, double-blind, randomized, placebo-controlled trial in patients with AOSD and active joint involvement. Randomization, stratified by pretreatment status with biological disease-modifying antirheumatic drugs (bDMARDs) and study center, was performed in a 1:1 ratio to the canakinumab or the placebo arm according to Atkinsons’ DA-optimal biased coin algorithm. In patients treated with nonsteroidal anti-inflammatory drugs (NSAIDs), glucocorticoids or conventional DMARDs, a stable dose prior to randomization and throughout study treatment was required (≥2 weeks (NSAIDs), ≥1 week (glucocorticoids with a dose of ≤10 mg/day prednisolone equivalent) and ≥6 weeks (conventional DMARDs)). Depending on the pharmacokinetic properties of the respective bDMARDs, a washout period between 1 week and 9 months was required [30]. Plasma from 31 adult-onset Still’s disease (AOSD) patients was analyzed. Seventeen of the patients (ten female and seven male) had been treated subcutaneously with canakinumab at a dose of 4 mg/kg body weight up to a maximum of 300 mg every 4 weeks. The other 14 patients (10 female and 4 male) had been randomized to placebo and used as a control group. Samples were analyzed at three time points: baseline week 0, at week 1 and at week 4. The CONSIDER study (CACZ885GDE01T) was conducted according to the ethical principles of the Declaration of Helsinki. The study protocol and all amendments were reviewed by the Independent Ethics Commission of the State of Berlin (Ethik-Kommission des Landes Berlin, LAGeSo), and Independent Ethics Committees for each center. An approval from the German regulatory authority was received prior to the start of the study. The reference number at the LAGeSo is: 11/0561-ZS EK 11. All patients provided written informed consent prior to the screening visit.

The additional collection of research samples for this biomarker analysis was a sub-project to the main study. The ethics committee also approved the additional patient consent and a separate signed patient consent form was used for all samples analyzed in this report.

A detailed description of patients analyzed in this study is shown in Table 1.

## 3. Methods

To investigate the exclusive effect of canakinumab on the expression of inflammatory cytokines, multiplex analysis and ELISA were used to test the concentrations of several cytokines in two patient groups (placebo versus canakinumab) at three time points (baseline week 0, week 1 and week 4) and multiple comparisons were applied between the two groups.

sCD163, IFN-α2 and BAFF/TNFSF13B were tested by Bio-Plex Pro Human Inflam 1 3plx EXP; IFN-γ, interleukin-1 receptor antagonist (IL-1RA), interleukin-2 receptor alpha (IL-2RA), IL-6, IL-10, IL-17, IL-18, MIG (CXCL 9), IP-10 (CXCL 10), and TNF-α were tested using Bio-Plex Pro Hu Screening Panel 10plx EXP, both from BioRad, Hercules, CA, USA. Soluble (s)L-Selectin and CXCL 13 were tested using Human sL-Selectin and Human CXCL 13 (BLC) ELISA Kits, respectively, both from Invitrogen, Waltham, Massachusetts, USA. Legend Max ELISA Kit Human MRP8/14 (Calprotectin) from Biolegend, San Diego, CA, USA was used to test MRP8/14 and levels of S100A12 were measured using Circulex S100A12/EN-RAGE ELISA Kit Ver.2 from MBL International, Woburn, MA, USA.

## 4. Statistics

As the primary outcome of the study, the 28-joint Disease Activity Score based on erythrocyte sedimentation rate (DAS28-ESR) was used to evaluate disease activity for all patients at week 0, week 1, week 4 and week 12. After discussion with the EMA, DAS28 was used as an established score in rheumatology to measure response with respect to arthritic manifestation. Of note, the Pouchot score was not fully validated at initiation of the CONSIDER study [31]. DAS28 ≤ 3.2 was interpreted as low, 3.2 < DAS28 ≤ 5.1 as moderate and DAS28 > 5.1 as high disease activity [32], whereas DAS28 < 2.6 corresponds to disease remission [33]. A good EULAR response to treatment was defined as an improvement of DAS28-ESR > 1.2 from baseline to week 12 (responders) [32]. For statistical analysis, logarithmic normally distributed data were used for all values that did not fully fit a normal distribution and extreme outliers were removed. Two-way repeated-measures analysis of variance (ANOVA) with Tukey’s multiple comparisons tests (95% confidence interval) and Sidak’s multiple comparisons test (95% confidence interval) was used to analyze whether time, treatment (placebo or canakinumab) or the interaction between both factors had a statistically significant effect on the expression of cytokines. The effect of the response rate (responder or non-responder) during treatment in canakinumab patients was analyzed in the same way. For all positive results, one-way repeated-measures ANOVA with Tukey’s multiple comparisons test (95% confidence interval) was applied to determine the simple main effect of time on the expression of cytokines in each treatment group. For responder and non-responder groups, repeated measures *t*-test was used. Spearman’s correlation coefficient was used to analyze the correlation between all analyzed cytokines and acute phase reactants, C-reactive protein (CRP) and ferritin. GraphPad Prism 7 software (San Diego, CA, USA) was used for all statistical analyses, and statistical significance was set at *p* < 0.05.

## 5. Results

### 5.1. Statistically Significant Differences in the Concentrations of MRP8/14 (Calprotectin), S100A12, IL-2RA, IL-6, IL-18 and sL-Selectin between Placebo and Canakinumab Groups

By two-way repeated-measures ANOVA, no significant differences were detected in the biomarkers CXCL13, BAFF, sCD163, IP-10, MIG, TNF-α and IL-1RA between the samples of patients treated with placebo or canakinumab.

**Figure 1:**
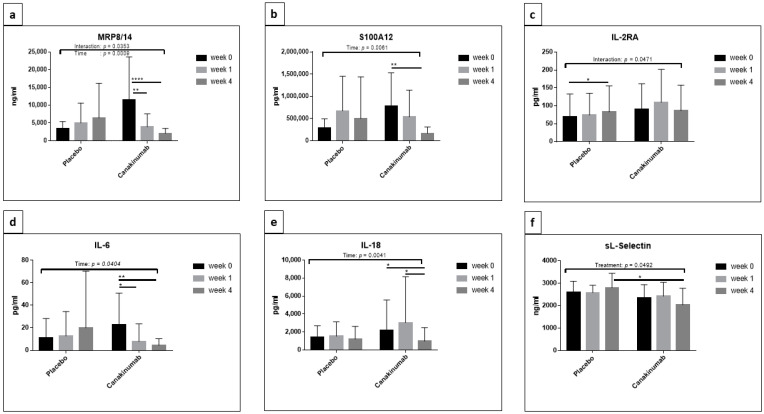
Representative cytokines that showed a significant reduction under the effect of canakinumab. A comparison of the concentrations of MRP8/14 (**a**), S100A12 (**b**), IL-2RA (**c**), IL-6 (**d**), IL-18 (**e**) and sL-Selectin (**f**) between placebo and canakinumab patients at week 0, week 1 and week 4 was applied. The bar plots represent the mean with SD. Two-way repeated-measures ANOVA with Tukey’s multiple comparisons test and Sidak’s multiple comparisons test was performed using GraphPad Prism 7 and statistical significance was set at *p* < 0.05. * *p* < 0.05, ** *p* < 0.01 and **** *p* < 0.0001.

Two-way repeated-measures ANOVA revealed a statistically significant effect of interaction between treatment type (placebo or canakinumab) and time (F (2, 30) = 3.744, *p* = 0.0353) on MRP8/14 concentrations. There was no difference between the two treatment groups with respect to MRP8/14 concentrations, but there was a statistically significant difference between time points (F (2, 30) = 8.983, *p* = 0.0009) between the two groups, with a significant reduction in MRP8/14 in the canakinumab group at week 4 (Figure 1a). An additional Tukey’s multiple comparisons test showed significant decreases between weeks 0 and 1 (adjusted *p* = 0.0065) and weeks 0 and 4 (adjusted *p* ≤ 0.0001) in the canakinumab group, while no significant differences could be detected in the placebo group.

**Figure 2:**
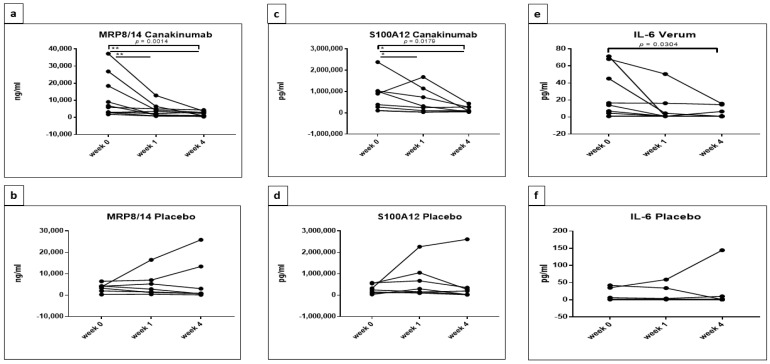
Confirmed significant reduction in MRP8/14 (**a**,**b**), S100A12 (**c**,**d**) and IL-6 (**e**,**f**) in canakinumab patients analyzing each group separately. One-way repeated-measures ANOVA with Tukey’s multiple comparisons test was used to investigate the effect of treatment (placebo or canakinumab) on the concentrations of cytokines during three time points (week 0, week 1 and week 4) in each group separately. GraphPad Prism 7 was used and statistical significance was set at *p* < 0.05. * *p* < 0.05, ** *p* < 0.01.

This result was further confirmed by applying one-way repeated-measures ANOVA for each treatment group separately with three time points. Canakinumab showed a significant decrease in MRP8/14 between weeks (*p* = 0.0014); Tukey’s multiple comparisons test showed significant reductions in MRP8/14 between weeks 0 and 1 (adjusted *p* = 0.0035) and weeks 0 and 4 (adjusted *p* = 0.0064) in the canakinumab group in contrast to the placebo (Figure 2a,b).

By applying two-way repeated-measures ANOVA on S100A12 concentrations, only a significant effect of time could be detected (F (2, 26) = 6.245, *p* = 0.0061); following this, a Tukey’s multiple comparisons test showed a significant decrease in S100A12 levels between weeks 0 and 4 (adjusted *p* = 0.0041) in the canakinumab group exclusively (Figure 1b). One-way repeated-measures ANOVA for each treatment group separately confirmed this result and showed a significant decrease in the concentrations of S100A12 in the canakinumab group only (*p* = 0.0179). The analysis between time points by Tukey’s multiple comparisons testing reflected a significant decrease between weeks 0 and 1 and between weeks 0 and 4 (*p* = 0.0417 and 0.0321, respectively) (Figure 2c,d).

The analysis of IL-2RA by two-way repeated-measures ANOVA demonstrated a significant interaction between treatment type and time (F (2, 30) = 3.388, *p* = 0.0471), with decreased expression in canakinumab at week 4. Neither time nor treatment type solely showed a significant effect on the concentrations of IL-2RA. An additional Tukey’s multiple comparisons test showed a significant increase between weeks 0 and 4 (adjusted *p* = 0.0429) in the placebo group, while no significant differences could be detected in canakinumab-treated patients (Figure 1c).

The concentrations of IL-6 differed significantly with respect to time (F (2, 32) = 3.555, *p* = 0.0404) in statistical analysis using a two-way repeated-measures ANOVA. As can be seen in Figure 1d, the concentrations of IL-6 decreased in patients treated with canakinumab, while they increased in patients treated with placebo. Additional Tukey’s multiple comparisons testing revealed a significant difference in patients treated with canakinumab, in which the concentrations of IL-6 decreased between weeks 0 and 1 (adjusted *p* = 0.0133) and weeks 0 and 4 (adjusted *p* = 0.008).

The significantly decreased concentrations of IL-6 over time in patients treated with canakinumab and not in the placebo group were further demonstrated by one-way repeated-measures ANOVA (*p* = 0.0304) (Figure 2e,f).

The two-way repeated-measures ANOVA of IL-18 concentrations revealed a significant difference with respect to time (F (2, 30) = 6.634, *p* = 0.0041), with a significant decrease in canakinumab at week 4. Further Tukey’s multiple comparisons testing revealed a significant decrease in the concentrations of IL-18 between week 0 and 4 (adjusted *p* = 0.0436) and between weeks 1 and 4 (adjusted *p* = 0.0118) only in canakinumab patients (Figure 1e). One-way repeated-measures ANOVA showed no significant differences of IL-18 in both groups.

Two-way repeated-measures ANOVA revealed a significant difference in concentrations of sL-selectin related to type of treatment (placebo or canakinumab) (F (1, 15) = 4.578), *p* = 0.0492), whereas increased concentrations of sL-selectin could be seen in the placebo group at week 4; an obvious decrease was observed only in canakinumab-treated patients. Comparison of the concentrations of sL-selectin between two groups at each time point by Sidak’s multiple comparisons test showed that the concentrations at week 4 were significantly decreased in canakinumab patients when compared to placebo (adjusted *p* = 0.0361) (Figure 1f). Further one-way repeated-measures ANOVA for each treatment group separately with three time points showed no significant differences in sL-selectin concentrations between different weeks in both groups.

IFN-γ, IL-10, IL-17A and IFN-α2 were below the lower detection limit of the multiplex assay (1.57, 1.06, 2.44 and 0.95 pg/mL, respectively); therefore, no statistical analyses were applied.

Detailed results of two-way and one-way repeated-measures ANOVA for significantly different cytokines are shown in Table 2 and Table 3, respectively.

### 5.2. Response Rate Has an Effect on the Concentrations of MRP8/14, S100A12, IL-1RA, IL-18 and IL-6 in Canakinumab Group

To analyze the effect of rate of response on the concentrations of cytokines, canakinumab patients were classified into responders and non-responders based on their DAS28-ESR improvement, and the concentrations of biomarkers were statistically analyzed. Since there were fewer samples at week 1, only concentrations at weeks 0 and 4 were evaluated. Two-way repeated-measures ANOVA showed no significant differences between responders and non-responders for the biomarkers CXCL13, BAFF, sCD163, MIG, IP-10, TNF-α, IL- 2RA, sL-selectin and IL-6.

**Figure 3:**
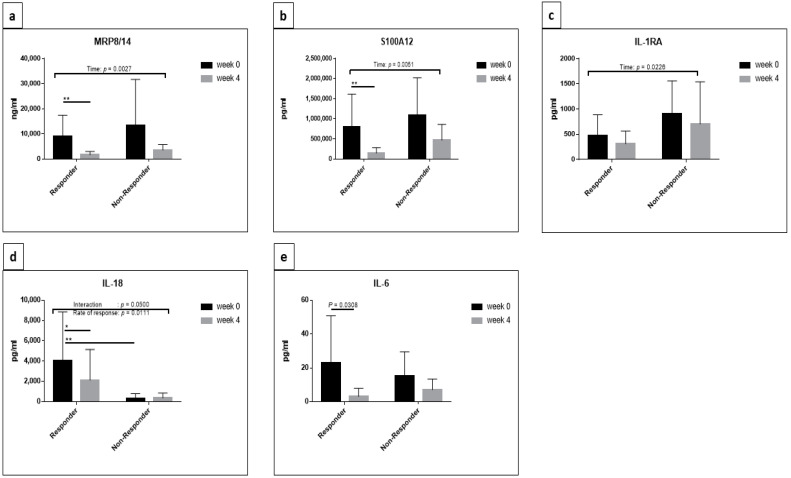
Representative cytokines that showed a significant reduction in response to canakinumab. A comparison of the expression of MRP8/14 (**a**), S100A12 (**b**), IL-1RA (**c**), IL-18 (**d**) and IL-6 (**e**) between responders and non-responders of canakinumab patients at week 0 and week 4 was applied. The bar plots represent the mean with SD. Two-way repeated-measures ANOVA with Tukey’s multiple comparisons test and Sidak’s multiple comparisons test was performed using GraphPad Prism 7 and statistical significance was set at *p* < 0.05. * *p* < 0.05, ** *p* < 0.01.

**Figure 4:**
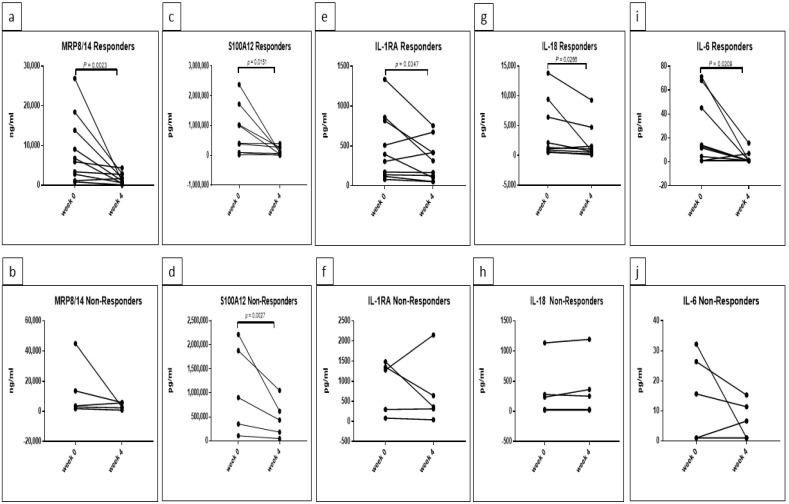
Confirmed significant reduction in MRP8/14 (**a**,**b**), S100A12 (**c**,**d**), IL-1RA (**e**,**f**), IL-18 (**g**,**h**) and IL-6 (**i**,**j**) in responders of canakinumab patients. Investigation of the effect of response rate in each responder and non-responder groups separately. Repeated-measures *t*-test was applied using GraphPad Prism 7 and statistical significance was set at *p* < 0.05.

For MRP8/14, two-way repeated-measures ANOVA determined a significant difference with respect to time (F (1, 13) = 13.61, *p* = 0.0027). Further Sidak’s multiple comparisons testing showed a significant decrease in responders between weeks 0 and 4 (adjusted *p* = 0.0023), as shown in Figure 3a. There was no significant decrease in concentrations of MRP8/14 in non-responders. By applying *t*-test for each group separately, a significant decrease in MRP8/14 between week 0 and week 4 could be detected in the responder group (*p* = 0.0023) exclusively (Figure 4a,b).

Two-way repeated-measures ANOVA detected significant differences in the concentrations of S100A12 with respect to time (F (1, 12) = 11.65 *p* = 0.0051); when applying comparison between time points by Sidak’s multiple comparisons test, a significant decrease in the concentrations of S100A12 could be detected in the responder group exclusively (adjusted *p* = 0.0056) (Figure 3b). Additional *t*-test in each group separately showed a significant decrease in responders and non-responders at week 4 (*p* = 0.0151 and 0.0027, respectively) (Figure 4c,d).

A significant effect of time was also observed for IL-1RA concentrations by two-way repeated-measures ANOVA (F (1, 13) = 6.688, *p* = 0.0226) (Figure 3c). Although a decrease was observed in both groups, no significant difference was determined by Sidak’s multiple comparisons test. *t*-test for each group revealed significant decrease in IL-1RA in responders exclusively (*p* = 0.0347) (Figure 4e,f).

Two-way repeated-measures ANOVA determined a significant effect of the interaction between time and response rate on the concentrations of IL-18 (F (1, 12) = 4.749, *p* = 0.05), with a significant decrease at week 4 detected by Sidak’s multiple comparisons test in the responder group exclusively (adjusted *p* = 0.0132). Two-way repeated-measures ANOVA also detected a significant effect regarding the rate of response (responders versus non-responders) (F (1, 12) = 8.989, *p* = 0.0111) with observable reduction in the concentrations of IL-18 in non-responders at both time points when comparing to responders. Sidak’s multiple comparisons testing showed a significant decrease in the concentrations of IL-18 in the non-responder group compared to the responder group at week 0 (adjusted *p* = 0.0042) (Figure 3d). Applying *t*-test for each group separately demonstrated a significant decrease in IL-18 at week 4 only in the responder group (*p* = 0.0266) (Figure 4g,h).

Although two-way repeated-measures ANOVA applied on IL-6 concentrations revealed no significant differences between responder and non-responders, further Sidak’s multiple comparisons testing showed significant reduced concentrations of IL-6 in responders at week 4 (adjusted *p* = 0.0308) (Figure 3e). The *t*-test confirmed the result and showed a significant decrease in responder group exclusively (*p* = 0.0209) (Figure 4i,j).

Detailed results of two-way repeated-measures ANOVA followed by *t*-test for significantly different cytokines are shown in Table 4 and Table 5, respectively.

### 5.3. Significant Correlations between Certain Cytokine/Chemokine Levels and CRP as well as Ferritin Were Detected at Week 4

**Figure 5:**
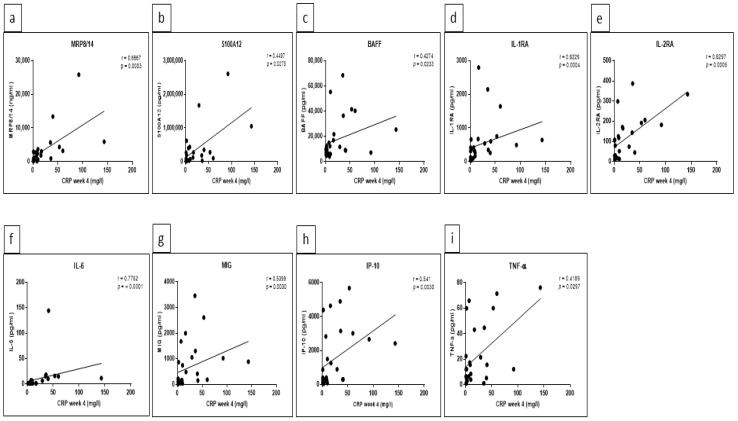
Significant correlations between cytokines/chemokines and C-reactive protein (CRP) at week 4. Spearman’s correlation coefficient r and *p* values of correlation analyses between CRP and MRP8/14 (**a**), S100A12 (**b**), BAFF (**c**), IL-1RA (**d**), IL-2RA (**e**), IL-6 (**f**), MIG (**g**), IP-10 (**h**), and TNF-α (**i**) in all patients. GraphPad Prism 7 was used and statistical significance was set at *p* < 0.05.

**Figure 6:**
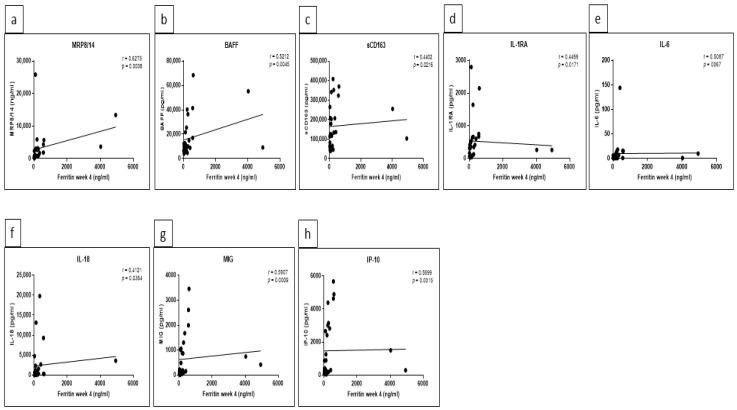
Significant correlations between certain cytokines/chemokines and ferritin at week 4. Spearman’s correlation coefficient r and *p* values of correlation analyses between ferritin and MRP8/14 (**a**), BAFF (**b**), sCD163 (**c**), IL-1RA (**d**), IL-6 (**e**), IL-18 (**f**), MIG (**g**) and IP-10 (**h**) in all patients. GraphPad Prism 7 was used and statistical significance was set at *p* < 0.05.

To analyze if cytokine/chemokine levels correlated with the activity of disease, Spearman’s correlation coefficient was calculated for acute phase reactants such as C-reactive protein (CRP) and ferritin. The analyses were applied in all patients as one group at baseline (week 0) and week 4. At baseline, a correlation with CRP was only observed for CXCL-13 and IL-6 (r values 0.4072 and 0.4368, with *p* values 0.035 and 0.0227, respectively). At week 4, we found significant correlations with CRP for MRP8/14 (r = 0.6667, *p* = 0.0003), S100A12 (r = 0.4497, *p* = 0.0275), BAFF (r = 0.4274, *p* = 0.0233), IL-1RA (r = 0.6229, *p* = 0.0004), IL-2RA (r = 0.6297, *p* = 0.0006), IL-6 (r = 0.7752, *p* = <0.0001), MIG (r = 0.5399, *p* = 0.003), IP-10 (r = 0.541, *p* = 0.003) and TNF-α (r = 0.4189, *p* = 0.0297) (Figure 5a–i), respectively. Likewise, ferritin levels also correlated with several additional cytokines at week 4 compared to baseline. In detail, significant correlations were observed for MRP8/14 (r = 0.6919, *p* = 0.0002), BAFF (r = 0.4537, *p* = 0.0175), IL-6 (r = 0.4808, *p* = 0.0129) and IL-8 (r = 0.4293, *p* = 0.0322) at baseline. In contrast, at week 4, a significant correlation was found with MRP8/14 (r = 0.6275, *p* = 0.0008), BAFF (r = 0.5212, *p* = 0.0045), sCD163 (r = 0.4402, *p* = 0.0216), IL-1RA (r = 0.4469, *p* = 0.0171), IL-6 (r = 0.5087, *p* = 0.0067), IL-18 (r = 0.4121, *p* = 0.0364), MIG (r = 0.5907, *p* = 0.0009) and IP-10 (r = 0.5699, *p* = 0.0015) (Figure 6a–h), respectively.

## 6. Discussion

In this study, we compared the inflammatory profile of well-characterized patients with AOSD treated with canakinumab to those treated with placebo in a controlled setting. For this purpose, we measured the concentrations of pro-inflammatory cytokines (IFN-α2, IFN-γ, TNF-α, BAFF, IL-6, IL-17 and IL-18), anti-inflammatory cytokine (IL-10), soluble cell adhesion molecule sL-selectin (CD62L), soluble CD163 (a monocyte/macrophage activation biomarker), alarmins (MRP8/14, S100A12) and chemokines (MIG (CXCL 9), IP-10 (CXCL10), and CXCL13). Additionally, we measured IL-1 receptor antagonist (IL-1RA), a natural receptor antagonist for IL-1 and efficient therapeutic molecule in AOSD, and soluble interleukin-2 receptor alpha (sIL-2RA), a marker of T-cell activation. We confirmed the effectiveness of canakinumab on modulating the concentrations of alarmins (MRP 8/14 and S100A12), IL-1RA, IL-18 and IL-6 in patients with AOSD with the strongest association with response rate. As known, alarmins seem to have an important function in AOSD pathogenesis; of those, calcium binding proteins MRP8/14 (S100A8/A9) and S100A12 showed to be useful markers of disease activity and severity in AOSD [34,35]. Our results showed that under treatment with canakinumab, concentrations of both S100 proteins were significantly reduced in AOSD patients when compared to placebo. Moreover, when we classified canakinumab patients by treatment response, MRP 8/14 showed a significant decrease in responders, and S100A12 was significantly decreased in responders and non-responders.

Moreover, a significant increase in the soluble form of IL-2RA at week 4 was also detected in the placebo group, whereas it decreased in canakinumab-treated patients. This is in agreement with previous studies that showed increased soluble IL-2RA in association with disease activity in chronic articular AOSD [23], and serum levels of sIL-2RA were significantly higher in patients with active versus inactive AOSD and decreased significantly with anti-inflammatory therapy [22]. IL-1RA is a natural inhibitor of the pro-inflammatory effect of IL-1; it binds IL-1 receptors without inducing a cellular response, thereby modulating a variety of interleukin-1-related immune and inflammatory responses [36]. IL-1RA levels are elevated in different diseases including auto-immune diseases [37,38]. Of note, it was elevated in AOSD patients [39] and significantly higher in patients with AOSD than SLE [40]. Although anakinra, a recombinant version of the interleukin-1 receptor antagonist is used in the treatment of AOSD [39,41], the increase in in vivo circulating IL-1RA levels corresponds to a delayed event in response to IL-1 production and may represent a preventive mechanism in excessive inflammatory response. Moreover, IL-1RA could be considered as an acute phase protein because its expression is regulated by pro-inflammatory cytokines in hepatocytes [42]. In our analysis, canakinumab, an IL-1β inhibitor, could significantly reduce levels of IL-1RA in responders, confirming that AOSD is an IL-1-mediated disease. Significantly higher levels of IL-6 were seen in both sera and skin tissues of patients with active AOSD when compared to healthy donors and quiescent AOSD patients, respectively [43]. Additionally, serum levels of free and total IL-18 were significantly higher in patients with AOSD than healthy donors and control patients with other diseases, including rheumatoid arthritis (RA), systemic lupus erythematosus (SLE) and psoriasis [40]. Furthermore, both cytokines (IL-6 and IL-18) showed an association with clinical activity and declined significantly in the remission phase [43]. This is consistent with the efficiency of canakinumab in significantly reducing the concentrations of both cytokines in our patients and more intensely in the responder group. Notably, the reduction effect of canakinumab on the concentrations of IL-1RA, IL-6, IL-18 and S100A12 in our cohort had been confirmed recently in a systemic juvenile idiopathic arthritis cohort. Moreover, in both cohorts, responders showed higher levels of IL-18 at baseline [44]. L-selectin is a cell adhesion molecule expressed on most leukocytes and is involved in their trafficking to sites of inflammation [14]. The level of soluble L-selectin is used as a surrogate biomarker for leukocyte activity triggered during different autoimmune diseases [45,46]. Our comparison between canakinumab and placebo patients showed a significant effect of canakinumab on lowering the concentrations of sL-selectin at week 4, and significant reduction could be detected in canakinumab when compared to the placebo group at week 4. Although the concentrations of TNF-α were higher in the active untreated AOSD patients when compared to healthy donors [43], canakinumab showed no effect on the concentrations of TNF cytokines used in this study (TNF-α and BAFF).

The concentrations of INF-γ [22], IL-17 [20] and IFN-α2 [21] and a classical anti-inflammatory cytokine IL-10 [25] were higher in AOSD patients than in healthy controls. In our patients, the levels of these cytokines were below the detection limit of the multiplex assay and this consistent with a previous study [40] that also showed undetectable levels of INF-γ, IL-17 and IFN-α. Despite the higher levels of CXCL10 and CXCL13 in AOSD than RA and healthy donors and the confirmed potential role of CXCL9, CXCL10 and CXCL13 as clinical biomarkers for disease activity in AOSD [47,48], our analysis showed no difference between concentrations of these CXC chemokine ligands before and after canakinumab treatment. Moreover, although higher levels of sCD163 in AOSD compared to healthy donors and correlation with disease activity were identified previously [49], no effect of canakinumab could be detected in our analysis. However, despite the robust observations in our study with a well-characterized cohort of patients, there are several limitations to consider. According to the inclusion and exclusion criteria and the outcome measurements as defined by the study protocol, our results are based on a selected cohort of patients with predominant articular disease manifestation. The sample size was small and the biomarker measurements were performed only at two time points over a short time period. Thus, further validations of our results are needed in another larger patient cohort over a longer observation period. Furthermore, it would be of utmost interest to investigate the same biomarker profile in patients with a predominant systemic manifestation of AOSD.

## 7. Conclusions

In this study, we provide evidence that canakinumab treatment in AOSD has a diverse impact on the cytokine profile of responding patients. Furthermore, we identified a potential biomarker profile for follow-up analyses consisting of S100 proteins, IL-2RA, IL1RA, IL-6 and IL-18 and sL-selectin.

## Figures and Tables

**Table 1:** Detailed characteristics of patients investigated in the study.

Patients			Placebo			Canakinumab		
			Men	Women	Total	Men	Women	Total
Number			4	10	14	7	10	17
Mean age in years (average)			35 (28–40)	46 (24–70)	40	41 (22–63)	45 (24–61)	43
CRP mg/L baseline mean			48	37	39	33	37	35
CRP mg/L week 12 mean			21	25	24	14	25	21
Ferritin ng/mL baseline mean			883	718	751	514	811	689
Ferritin ng/mL week 12 mean			322	633	576	278	548	437
Medication before study	c-DMARDs		2	5	7	6	7	13
	biological	Anti-IL1 (anakinra)	2	8	10	6	5	11
		Anti-TNF		4	4	1	2	3
		Anti-IL6		2	2	2	2	4
	steroidal	Prednisolone	2	9	11	7	10	17
	NSAIDs	COXIB				2	2	4
DAS28-ESR baseline	Remission							
	Low			1	1			
	Moderate		1	3	4	5	6	11
	High		1	5	6	2	2	4
DAS28-ESR week 12	Remission			2	2	2	3	5
	Low			3	3	1	2	3
	Moderate		1	3	4	4	2	6
	High		1	1	2		1	1
DAS28-ESR Improvement by at least 1.2	Yes			5	5	4	6	10
	No		2	4	6	3	2	5

CRP: C-reactive protein, NSAIDs: nonsteroidal anti-inflammatory drugs, c-DMARDs: conventional disease-modifying antirheumatic drugs, DAS28-ESR: disease activity score uses 28 joint counts—erythrocyte sedimentation rate.

**Table 2:** Detailed results of two-way repeated-measures ANOVA with Tukey’s and Sidak’s multiple comparisons tests for placebo and canakinumab groups. * *p* < 0.05, ** *p* < 0.01, *** *p* < 0.001 and **** *p* < 0.0001.

Cytokine	Source of Variation	*p* Value	Summary	F (DFn, DFd)	Tukey’s Multiple Comparisons Test	Adjusted *p* Value	Summary
MRP8/14	Interaction	0.0353	*	F (2, 30) = 3.744	Placebo		ns
	Treatment	0.5932	ns		Canakinumab		
	Time	0.0009	***	F (2, 30) = 8.983	week 0 vs. week 1	0.0065	**
					week 0 vs. week 4	<0.0001	****
					week 1 vs. week 4	0.157	ns
S100A12	Interaction	0.0757	ns		Placebo		ns
	Treatment	0.8699	ns		Canakinumab		
	Time	0.0061	**	F (2, 26) = 6.245	week 0 vs. week 1	0.2236	ns
					week 0 vs. week 4	0.0041	**
					week 1 vs. week 4	0.1746	ns
IL-2RA	Interaction	0.0471	*	F (2, 30) = 3.388	Placebo		
	Treatment	0.6039	ns		week 0 vs. week 1	0.0972	ns
	Time	0.086	ns		week 0 vs. week 4	0.0429	*
					week 1 vs. week 4	0.9209	ns
					Canakinumab		ns
IL-6	Interaction	0.1085	ns		Placebo		ns
	Treatment	0.7974	ns		Canakinumab		
	Time	0.0404	*	F (2, 32) = 3.555	week 0 vs. week 1	0.0133	*
					week 0 vs. week 4	0.008	**
					week 1 vs. week 4	0.9779	ns
IL-18	Interaction	0.6183	ns		Placebo		ns
	Treatment	0.9114	ns		Canakinumab		
	Time	0.0041	**	F (2, 30) = 6.634	week 0 vs. week 1	0.8451	ns
					week 0 vs. week 4	0.0436	*
					week 1 vs. week 4	0.0118	*
					Sidak’s multiple comparisons test	Adjusted *p* Value	Summary
sL-Selectin	Interaction	0.2684	ns		Placebo-Canakinumab		
	Treatment	0.0492	*	F (1, 15) = 4.578	week 0	0.8361	ns
	Time	0.6646	ns		week 1	0.9326	ns
					week 4	0.0361	*

**Table 3:** Detailed results of one-way repeated-measures ANOVA with Tukey’s multiple comparisons test for placebo and canakinumab separately for three time points. * *p* < 0.05, ** *p* < 0.01.

Cytokine	Group	*p* Value	Summary	Tukey’s Multiple Comparisons Test	Adjusted *p* Value	Summary
MRP8/14	Placebo	0.3967	ns	Placebo		ns
	Canakinumab	0.0014	**	Canakinumab		
				week 0 vs. week 1	0.0035	**
				week 0 vs. week 4	0.0064	**
				week 1 vs. week 4	0.0758	ns
S100A12	Placebo	0.1282	ns	Placebo		ns
	Canakinumab	0.0179	*	Canakinumab		
				week 0 vs. week 1	0.0417	*
				week 0 vs. week 4	0.0321	*
				week 1 vs. week 4	0.2709	ns
IL-6	Placebo	0.6997	ns	Placebo		ns
	Canakinumab	0.0304	*	Canakinumab		ns

**Table 4:** Detailed results of two-way repeated-measures ANOVA with Sidak’s multiple comparisons test for responders and non-responders. * *p* < 0.05, ** *p* < 0.01.

Cytokine	Source of Variation	*p* Value	Summary	F (DFn, DFd)	Sidak’s Multiple Comparisons Test	Adjusted *p* Value	Summary
MRP8/14	Interaction	0.2892	ns		week 0–week 4		
	DAS28 improvement	0.3022	ns		Responder	0.0023	**
	Time	0.0027	**	F (1, 13) = 13.61	Non-Responder	0.2562	ns
S100A12	Interaction	0.3104	ns		week 0–week 4		
	DAS28 improvement	0.1944	ns		Responder	0.0056	**
	Time	0.0051	**	F (1, 12) = 11.65	Non-Responder	0.3075	ns
IL-1RA	Interaction	0.9816	ns		week 0–week 4		
	DAS28 improvement	0.3475	ns		Responder	0.0877	ns
	Time	0.0226	*	F (1, 13) = 6.688	Non-Responder	0.2501	ns
IL-18	Interaction	0.05	*	F (1, 12) = 4.749	week 0–week 4		
	DAS28 improvement	0.0111	*	F (1, 12) = 8.989	Responder	0.0132	*
	Time	0.108	ns		Non-Responder	0.9548	ns
					Responder-Non-Responder		
					week 0	0.0042	**
					week 4	0.0523	ns
IL-6	Interaction	0.275	ns		week 0–week 4		
	DAS28 improvement	0.5756	ns		Responder	0.0308	*
	Time	0.0585	ns		Non-Responder	0.8209	ns

**Table 5:** Detailed results of *t*-test between week 0 and week 4 for responder and non-responder groups separately. * *p* < 0.05, ** *p* < 0.01.

Cytokine	Group	*p* Value	Summary
MRP8/14	Responders	0.0023	**
	Non-Responders	0.196	ns
S100A12	Responders	0.0151	*
	Non-Responders	0.0027	**
IL-1RA	Responders	0.0347	*
	Non-Responders	0.2558	ns
IL-18	Responders	0.0266	*
	Non-Responders	0.3871	ns
IL-6	Responders	0.0209	*
	Non-Responders	0.601	ns

## Data Availability

The datasets used and/or analyzed for the current study are available from the corresponding author on reasonable request.
